# The Antidepressant-Like Effects of Hesperidin in Streptozotocin‐Induced Diabetic Rats by Activating Nrf2/ARE/Glyoxalase 1 Pathway

**DOI:** 10.3389/fphar.2020.01325

**Published:** 2020-08-28

**Authors:** Xia Zhu, Haiyan Liu, Yuan Liu, Yajing Chen, Yaowu Liu, Xiaoxing Yin

**Affiliations:** Jiangsu Key Laboratory of New Drug Research and Clinical Pharmacy, Xuzhou Medical University, Xuzhou, China

**Keywords:** depression, diabetes, hesperidin, glyoxalase 1, AGEs/RAGE axis, Nrf2/ARE pathway

## Abstract

The co-occurrence of diabetes and depression is a challenging and underrecognized clinical problem. Alpha-carbonyl aldehydes and their detoxifying enzyme glyoxalase 1 (Glo-1) play vital roles in the pathogenesis of diabetic complications, including depression. Hesperidin, a naturally occurring flavanone glycoside, possesses numerous pharmacological properties, but neuroprotection by hesperidin in depression-like behaviors in diabetes was not observed. This study aimed to investigate the mechanisms and signaling pathways by which hesperidin regulates depression-like behaviors in diabetic rats and to identify potential targets of hesperidin. Rats with streptozotocin-induced diabetes were treated orally with hesperidin (50 and 150 mg/kg) or the nuclear factor erythroid 2-related factor 2 (Nrf2) inducer tert-butylhydroquinone (TBHQ, 25 mg/kg) for 10 weeks. After behavioral test, the brains were collected to evaluate the effects of hesperidin on Glo-1, Nrf2, protein glycation, and oxidative stress. Hesperidin showed antidepressant and anxiolytic effects in diabetic rats, as evidenced by the decreased immobility time in the forced swimming test, increased time spent in the center area of the open field test, and increased percentage of open-arm entries and time spent in the open arms in the elevated plus maze, as well as by the enhancement of Glo-1 and the inhibition of the AGEs/RAGE axis and oxidative stress in the brain. In addition, hesperidin caused significant increases in the Nrf2 levels and upregulated γ-glutamylcysteine synthetase, a well-known target gene of Nrf2/ARE signaling. *In vitro*, the effects of hesperidin on N2a cell injury caused by high glucose (HG) was assessed by MTT and LDH, and the effects on Nrf2 signaling were also assessed. We found that the Nrf2 inhibitor ML385 reversed the protective effects of hesperidin on the cell injury induced by HG. Hesperidin prevented the HG-induced reduction in the Nrf2 and Glo-1 levels, and ML385 reversed the effects of hesperidin on the expression of the proteins mentioned above, indicating that Nrf2 signaling is involved in the hesperidin-induced neuroprotective effects. Our findings indicate that the effects of hesperidin on ameliorating the depression- and anxiety-like behaviors of diabetic rats, which are mediated by the enhancement of Glo-1, may be due to the activation of the Nrf2/ARE pathway.

## Introduction

The co-occurrence of depression and diabetes has been recognized as an emerging global challenge (Fisher et al., 2012). Clinical studies have shown an overall two-fold increase in the prevalence of depression and anxiety in patients with type 1 and type 2 diabetes compared with that in the general population worldwide, and depression and anxiety adversely affect the quality of life and outcomes of diabetes patients (Grigsby et al., 2002; Hermanns et al., 2005; Roy and Lloyd, 2012). The interaction of depression and diabetes is bidirectional; depression adversely impacts the course of diabetes, and diabetes complications increase the risk of, and worsen the course of, depression (Semenkovich et al., 2015). Rats with streptozotocin-induced diabetes presented depression-like behaviors in the forced swimming test (FST), which is a predictive animal model of depression (Gomez and Barros, 2000; El-Marasy et al., 2014). However, the cause of depression in diabetes patients remains unclear.

Numerous studies have already shown that oxidative stress plays a major role in the pathogenesis of both types of diabetes mellitus and complications of diabetes (Ceriello et al., 2000; Fang et al., 2018). Methylglyoxal (MG), an alpha-carbonyl aldehyde, is one of the most powerful protein glycation agents that generates the majority of advanced glycation end-products (AGEs), which can increase oxidative stress levels (Sena et al., 2012). The major function of the glyoxalase system is the detoxification of MG and other reactive aldehydes, and glyoxalase 1 (Glo-1) is the main rate-limiting enzyme. In diabetic encephalopathy, the elevated levels of MG during hyperglycemia and the downregulation of Glo-1 induced by oxidative stress lead to a clear increase in advanced glycation end-products (AGEs) (Liu et al., 2013a; Zhu et al., 2018). Reduced glutathione (GSH), a cofactor required by Glo-1 to clear MG, is a main product of γ-glutamylcysteine synthetase (γ-GCS), which is regulated by the nuclear factor erythroid 2-related factor 2 (Nrf2)/antioxidant response element (ARE) signaling pathway. Mounting evidence suggests that Glo-1 overexpression has a therapeutic effect on diabetic complications (Maher et al., 2011; Liu et al., 2017b; Peng et al., 2017), including diabetic encephalopathy (Liu et al., 2013a; Zhu et al., 2018). MG formation and decreased Glo-1 function play crucial roles in the metabolism of chronic hyperglycemia (Thornalley, 1994); therefore, the induction or enhancement of Glo-1 is considered to be an important strategy for the prevention and treatment of common diabetic complications, such as depression.

Hesperidin (3,5,7-trihydroxy flavanone-7-rhamnoglucoside), a naturally occurring flavanone glycoside, is common in citrus species, including sweet oranges and lemons. According to reports, hesperidin and its aglycone, hesperetin, have numerous pharmacological properties, including antioxidative, anti-inflammatory, and antidiabetic properties (Parhiz et al., 2015; Alu’datt et al., 2017). Furthermore, hesperidin can alleviate diabetic neuropathy (Visnagri et al., 2014) and nephropathy (Zhang et al., 2018). Previous studies also suggested that treatment with hesperidin had antidepressant-like effects on rodents (Antunes et al., 2014; Feng et al., 2016; Fu et al., 2019). Hesperidin showed antidepressant-like properties and may be an interesting source of therapeutic agents for the treatment of depression (Souza et al., 2013; Donato et al., 2014). Most importantly, hesperetin worked as a small-molecule inducer of Glo-1, improving metabolic and vascular health in overweight and obese subjects (Xue et al., 2016). Moreover, the antioxidant activity of hesperidin was not limited to its free radical scavenging activity, but it also enhanced the cellular antioxidant defenses *via* the ERK/Nrf2 pathway (Chen et al., 2010; Elavarasan et al., 2012). Our previous report indicated that the downregulation of Glo-1 was associated with the inactivation of the Nrf2/ARE signaling pathway in neurons cultured in high-glucose conditions (Liu et al., 2017b). In addition, hesperidin or hesperetin can activate the Nrf2/ARE signaling pathway and increase the messenger RNA (mRNA) and protein levels of downstream target genes in some diseases (Chen et al., 2010; Ren et al., 2016). However, there is no report on whether hesperidin can improve depression in diabetes *via* the enhancement of Glo-1 function and the activation of the Nrf2/ARE signaling pathway.

Therefore, this study aims to examine the antidepressant- and anxiolytic-like effects of hesperidin in rats with type 1 diabetes, to investigate the induction of Glo-1 by hesperidin in the brains of diabetic rats, and to clarify the critical role of the Nrf2/ARE pathway in the enhancement of Glo-1 and the antidepressant and anxiolytic effects of hesperidin.

## Material and Methods

### Animals

Adult male Sprague Dawley rats weighing 200–220 g were obtained from the Experimental Animal Center, Xuzhou Medical University (Xuzhou, China). The rats were housed in groups of five in a room with controlled temperature (24 ± 1°C) and humidity (50 ± 10%) and a 12/12 h day/night cycle (lights on 7:00 a.m.), and the rats were given free access to water and food. All the animal experiments were carried out according to the license by Jiangsu Province Science and Technology Office (Nanjing, China). The housing, handling, and experimental procedures complied with the recommendations set forth by the Xuzhou Medical University Committee on Animals Care and Use. All efforts were made to minimize animal suffering.

### Experimental Design

Streptozotocin (STZ, Sigma-Aldrich, USA) was used to establish a model of type 1 diabetes, and it was injected intraperitoneally into the rats after they were fasted for 12 h (60 mg/kg, freshly dissolved in 0.1 mol/L sodium citrate buffer at pH 4.5). Age-matched normal rats were administered the same volume of citrate buffer alone. After 5 days, venous blood was collected to measure the fasting blood glucose (FBG) level using a reagent kit (Jiancheng Bioengineering Institute, Nanjing, China). FBG levels higher than 13.9 mmol/L indicated the successful establishment of the diabetes model, and rats meeting this criterion were randomly distributed into four groups. Each group received different doses of hesperidin (50 mg/kg, Hsd-L, and 150 mg/kg, Hsd-H), a representative inducer of Nrf2, namely, tert-butylhydroquinone (TBHQ, 25 mg/kg), or 1% carboxymethylcellulose sodium (CMC-Na) solution (DM). In addition, age-matched rats were divided into three groups: the control group, low-dose hesperidin (50 mg/kg, Hsd-L) group, and high-dose hesperidin (50 mg/kg, Hsd-L) group. Hesperidin (purity > 98%, Chengdu Okay Pharmaceutical Co. Ltd., China) was dissolved in 1% CMC-Na solution, while TBHQ (purity > 99%, Shanghai Sigma-Aldrich Corporation, China.) was dissolved in distilled water and freshly prepared each time. The rats received the relevant drug treatment by oral administration daily for 10 weeks. The age-matched and diabetes groups were given the same treatment without drug. We monitored the body weights weekly and the blood glucose levels monthly after the treatments. Thirty minutes after the final drug administration, behavioral performances were assessed by the FST, open field test (OFT), and elevated plus maze (EPM) on days 71, 72, and 73, respectively. After behavioral testing, all the rats were euthanized under ketamine anesthesia (100 mg/kg) administered intraperitoneally (Ambrogi Lorenzini et al., 1997) after 30 min of receiving the drugs, and blood samples were collected for the detection of corticosterone. Four brain tissues of the rats from each group were randomly selected for HE staining and immunohistochemistry assays, and the rest were used for biochemical analysis. The hippocampus and amygdala of rats were dissected according to landmarks identified by Paxinos and Watson (Paxinos and Watson, 1986). Dissected tissues from each hemisphere of both the brain regions were placed into separate prelabeled Eppendorf tubes and flash frozen in liquid nitrogen. All samples were stored at 80°C until further processing.

### Behavioral Testing

All the behavior tests were conducted between 09:00 and 14:00 h in a separated testing room with a light intensity of ~15 W. The tests were video-recorded using a video camera recorder (SANS Biological Technology Co., Ltd., Jiangsu, China). The data were collected and analyzed with any-maze behavioral tracking software (Stoelting, CO, USA). Behavioral measures (see below) were recorded by a trained observer who was unaware of the treatment condition. There were 11 rats in TBHQ group and 10 rats in other groups.

#### Forced Swimming Test

The forced swim test (FST) was carried out as previously described with minor modifications (Mizoguchi et al., 2011; Nasu et al., 2019). The rats were individually placed in a glass cylinder (height: 50 cm; diameter: 20 cm; water depth: 35 cm; water temperature: 24 ± 1°C) for 6 min. The duration of immobility was recorded during the last 5 min of the 6-min testing period. A rat was defined to be immobile when it performed only small movements to keep its head above water.

#### Open Field Test

The purpose of this test was to observe the general locomotive activity and anxiety-like behaviors of the rats. The rats were individually placed in the center of a square field surrounded by opaque, plastic walls (100 cm × 100 cm × 40 cm box). The rats freely explored the field for 5 min and were placed individually in one corner of the open field. The entry latency and time spent in the center area were recorded. The arena was cleaned with water between tests.

#### Elevated Plus Maze

The elevated plus maze (Huaibei Zhenghua Instruments, China) consisted of a maze with two open arms (60 × 10 cm) crossed by two closed arms (50 × 10 × 40 cm), and free access to all arms was available from the central platform (10 × 10 cm). The maze was elevated 80 cm from the floor. The rats were placed on the central platform facing an open arm. The number of entries into and the time spent in the open arms were recorded during the first 5 min. An entry was defined as all four paws in an arm. Anxiolytic effects were inferred from increases in the percentage of entries into the open arms and the percentage of time spent in the open arms.

### Determination of the Corticosterone Levels in Serum by ELISA

One day after the behavioral tests were completed, the rats were euthanized after 30 min of drug administration under ketamine anesthesia, and eight blood samples were collected for corticosterone analysis. The levels of corticosterone in the serum were determined using rat corticosterone ELISA kits (Boyun Biotechnology Co., Shanghai, China), according to the manufacturer’s instructions.

### Hematoxylin–Eosin (HE) Staining

Serial coronal sections (4-μm thick) were stained with hematoxylin and eosin (HE). Four tissue segments per group were prepared, and the histological profiles were explained. The CA1 subfield in the hippocampus of each brain section was examined using microscopy (Olympus BX43F, Japan) and an Image-Pro Plus 4.0 analysis system (Media Cybernetics, Silver Spring, MD). Cells with a distinct nucleus and nucleolus were regarded as intact neurons.

### Immunohistochemistry Analysis

A mouse polyclonal antibody against rat Nrf2 (Abcam, UK) was used for immunohistochemical staining of the brain. Four tissue segments per group were prepared, and immunohistochemical staining was carried out using the standard method as previously described (Liu et al., 2010). The immunohistochemistry two-step detection kit and poly-horseradish peroxidase-coupled anti-mouse IgG antibody (Zhongshan Golden Bridge Biotech Co., Beijing, China) were used in this assay. The quantitative analysis was performed in a blinded manner with a research microscope (Olympus BX43F). Five representative images were randomly selected in each slide. The number of Nrf2-positive cells in ten random microscopic fields was counted under 400× magnification. The images of Nrf2 immunostaining were analyzed with Image-Pro Plus 4.0 software to obtain signal intensity and area for each signal (Jensen, 2013).

### Determination of the Advanced Glycation End-Product Levels in the Brain Using a Fluorescence Assay

The AGE levels in the brains of the diabetic rats were detected through fluorospectrophotometry according to our previous report (Liu et al., 2013a). Each group contained seven samples. The AGE levels in the amygdala and hippocampus are presented as the enzyme activity of type I collagenase (U) per milligram protein.

### Detection of the Glo-1 Activity in the Brain by Ultraviolet Spectrophotometry

Seven samples were prepared, and the Glo-1 activity was measured by a spectrophotometric method that monitored the increase in the absorbance at 240 nm due to the formation of S-D-lactoylglutathione for 2 min at 25°C (Maher et al., 2011). The assay protocol was described in a previous report (Parhiz et al., 2015). The activity of Glo-1 was shown as the percentage of the production of S-D-lactoylglutathione per minute per milligram protein in normal rats (100%).

### Western Blot Analysis

Six amygdala and hippocampus were preserved after weighing and were homogenized with radioimmunoprecipitation assay (RIPA) lysis buffer containing protease and phosphatase inhibitors. The homogenates were centrifuged at 12,000 rpm for 15 min at 4°C. Western blotting was carried out using the standard method as previously described (Zhu et al., 2018). The primary antibodies used were as follows: anti-Glo-1, 1:1,000 (Abcam, UK); anti-RAGE, 1:1,000 (Abcam, UK); anti-Nrf2, 1.5:2,000 (Abcam, UK); anti-γ-GCS 1:1,000 (Bioworld Technology Inc., USA); and β-actin 1:1,000 (Bioworld Technology Inc., USA).

### Real-Time Quantitative PCR Assay

Seven samples in each group were prepared, and RNA extraction and real-time quantitative PCR (qPCR) were performed as previously described (Liu et al., 2013b). The primer sequences were as follows: Glo-1 (forward 5′-GCCTCTAAGCCAGACCACAT-3′, reverse 5′- GCAGCACTCAAAGCCATAAC-3′), RAGE (forward 5′-GGAAGGACTGAAGCTTGGAAGG-3′, reverse 5′- TCCGATAGCTGGAAGGAGGAGT-3′), γ-GCS (forward 5′- GAA GATGACGAGACGCAGAGTTAC -3′, reverse 5′-CAGGATCTTGAACGAA CGCCAGAC-3′), and β-actin (forward 5′- CCCATCTATGAGGGTTACGC -3′, reverse 5′- TTTAATGTCACGCACGATTTC -3′). Lower Ct values indicated higher amounts of PCR products. The data were normalized to β-actin and expressed as 2^−⊿⊿Ct^ values (⊿Ct = Ct_target_ – Ct_reference_).

### Determination of Oxidative Stress Indicators in the Brain

The reactive oxygen species (ROS), malondialdehyde (MDA), and GSH levels and the SOD activity in the amygdala and hippocampus were measured in each group by using of seven rats. A spectrophotometer using kits from JieMei Bioengineering Institute (Shanghai, China) was selected in this test according to the manufacturer’s instructions.

### Cell Culture and Drug Treatment

Mouse neuroblastoma neuro‐2a (N2a) cells were routinely maintained in Dulbecco’s modified Eagle medium (DMEM) (Gibco) containing 10% fetal bovine serum (FBS, Gibco), 10 U/ml penicillin, and 10 U/ml streptomycin at 37°C in a humidified atmosphere with 5% CO_2_. The N2a cells were divided into eight different groups: the normal glucose group (NG, 25 mmol/L glucose), high-glucose group (HG, 100 mmol/L glucose), osmotic pressure control group (MA, NG + 75 mmol/L mannitol), solvent control group (DMSO, HG + 0.1% dimethyl sulfoxide), low-dose hesperidin group (Hsd-L, HG + 5 μmol/L of hesperidin), middle-dose hesperidin group (Hsd-M, HG + 10 μmol/L of hesperidin), high-dose hesperidin group (Hsd-H, HG + 20 μmol/L of hesperidin), and Nrf2 inhibitor group (ML385, HG + 10 μmol/L of ML385). Hesperidin or ML385 (Selleck Chemicals, USA) was dissolved or diluted with DMSO and added when the cell culture medium was changed from NG to HG. After treatment for 48 h, the associated parameters were measured. The data were obtained by performing three independent experiments, each of which were performed in triplicate.

### Cell Viability Determination by MTT Assay

To examine the neuroprotective effect of hesperidin against high-glucose injury, N2a cells (1 × 10^5^ cells/well) were plated in 96‐well plates and treated with different doses of hesperidin or ML385. ML385 was administered 30 min after hesperidin treatment. The cell viability was estimated by using the 3‐4,5‐dimethyl thiazol‐2‐yl‐2,5‐diphenyltetrazolium bromide dye (MTT, Solarbio Life Sciences, China). MTT solution was added to each well according to the manufacturer’s instructions, and the plates were incubated for 4 h at 37°C. DMSO was added to each well to dissolve the formazan. The viability was then measured by evaluating the absorbance at 490 nm. Normal cells were used as the control group, and the cell viability of the control group was assumed to be 100%.

### Cell Death Determined by Lactate Dehydrogenase Assay

Previous findings have shown that the activity of the LDH released from damaged cells is proportional to the number of damaged neurons (McCord and Fridovich, 1969; Wang et al., 2015). After exposure to the drugs, 50 μl of the medium was removed, and the amount of LDH released from the cells was determined using a LDH kit (Solarbio Life Sciences, China) according to the manufacturer’s instructions. The absorbance of the samples was read spectrophotometrically at 450 nm. The results are expressed as the percentage of LDH released relative to that released from the control cells.

### Immunofluorescence Analysis

Cells plated on coverslips were fixed with 4% paraformaldehyde for 15 min, permeabilized with 0.5% Triton X-100 in PBS for 5 min, treated with blocking medium (1% bovine serum albumin in PBS) for 10 min, and then incubated with an anti-Nrf2 antibody (Abcam, Cambridge, UK) at 4°C overnight. The immune-reacted primary Nrf2 antibody was detected following a 1-h incubation in the dark at 37°C with a secondary antibody conjugated with DyLight 594 (EarthOx, Millbrae, CA, USA). The cells were then stained with 4′,6-diamidino-2-phenylindole (DAPI) (Vector, Burlingame, CA, USA) for 2 min in the dark at room temperature and washed. Then, the cells were mounted onto microscope slides in mounting medium. Observations were carried out using an Olympus BX43F fluorescence microscope.

### Statistical Analysis

All the data were analyzed using GraphPad Prism 6.0 software. All results were presented as the mean ± SEM. Data obtained from behavioral tests including FST, OFT, and EPM were analyzed by two-way analysis of variance (ANOVA) with Tukey’s *post hoc* test. All the other results were performed using one-way ANOVA with Dunnett’s *post hoc* test. The significance level was set to *p <* 0.05.

## Results

### Effects of Hesperidin on the Fasting Blood Glucose Levels and Body Weights of Diabetic Rats

The FBG levels of all the rats were measured at week 0 and week 10 after long-term treatment with hesperidin. The FBG levels began to rise after the injection of SD rats with STZ and remained elevated in the diabetic rats (*p <* 0.001) compared with the age-matched normal rats throughout the treatment process. However, hesperidin did not change the FBG levels of the diabetic rats at the late stage (week 10, **Table 1**). The body weights of the rats were matched between the groups at the beginning of the experiment. After 10 weeks after STZ injection, the body weight of the diabetic rats was significantly decreased compared with that of the age-matched normal rats (*p <* 0.001). The diabetic rats treated with hesperidin showed no significant difference in body weight compared with the diabetic rats without drug intervention (**Table 1**). The Nrf2 activator TBHQ did not show any significant effects on either the FBG levels or body weights of the diabetic rats.

**Table 1:** Effects of hesperidin and hesperetin on fasting blood glucose (FBG) and body weight in streptozotocin (STZ)-induced diabetic rats after treatment.

Groups	Fasting blood glucose (mmol/L)		Body weight (g)	
	Week 0	Week 10	Week 0	Week 10
Cont.	5.77 ± 0.19	6.64 ± 0.41	201.5 ± 2.4	323.6 ± 8.6
DM	17.00 ± 1.05^***^	29.81 ± 1.30^***^	200.5 ± 2.1	201.9 ± 2.8^***^
DM+Hsd-L	15.93 ± 0.94	29.97 ± 0.77	195.1 ± 3.5	212.8 ± 7.2
DM+Hsd-H	16.78 ± 1.01	29.81 ± 1.13	192.0 ± 4.4	203.0 ± 9.1
DM+TBHQ	15.90 ± 1.07	29.05 ± 3.99	199.1 ± 3.5	196.5 ± 8.6

Mean ± SEM, n=10-11.

^***^p < 0.001, vs. Cont. group.

### Hesperidin Exerted Antidepressant-Like and Anxiolytic-Like Effects on Diabetic Rats

**Figure 1:**
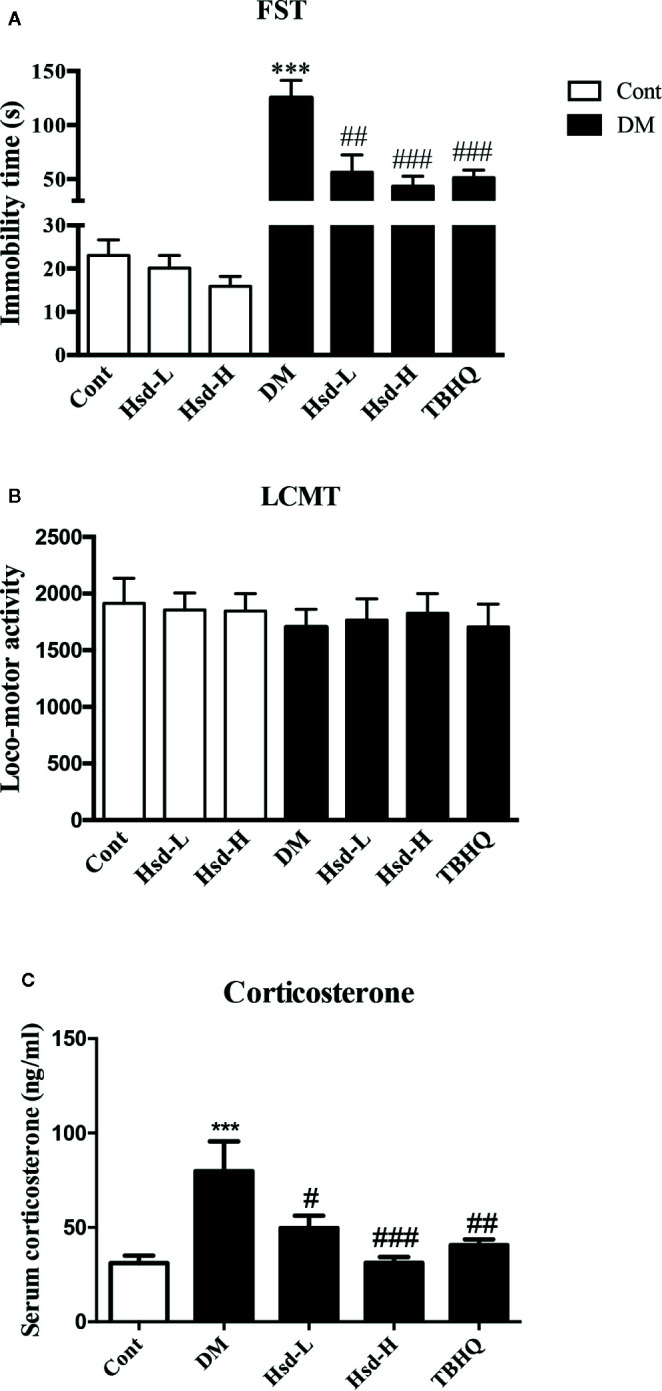
**(A)** Effects of hesperidin on the depression-like behaviors of diabetic rats in the forced swimming test (FST). **(B)** Effects of hesperidin on spontaneous locomotor activity in diabetic rats subjected to the locomotor activity test (LCMT). **(C)** Effects of hesperidin on serum corticosterone levels in diabetic rats. Cont, DM, Hsd-L, Hsd-H, and tert-butylhydroquinone (TBHQ) represent the control rats, STZ-induced diabetic rats (DM), control or diabetic rats treated with hesperidin at low and high (50, 150 mg/kg) doses, and diabetic rats treated with the Nrf2 activator TBHQ (25 mg/kg), respectively. The results are presented as the mean ± S.E.M., n = 10–11 (FST and LCMT) or n = 7–8 (corticosterone). ^***^p < 0.001, *vs*. Cont group; *^#^p* < 0.05, *^##^p* < 0.01, *^###^p* < 0.001, *vs*. DM group.

Depression-like behaviors were observed in the FST. Two‐way ANOVA of the immobility time in FST showed significant values for interactions [F(2,54) = 7.71, *p <* 0.001], hesperidin treatment factor [F(2, 54) = 10.89, *p* < 0.001], and diabetic factor [F(1, 54) = 45.49, *p <* 0.001]. As shown in **Figure 1A**, Tukey’s multiple comparisons test revealed that the duration of immobility of the diabetic rats was significantly increased compared with that of the normal rats (*p <* 0.001). However, chronic treatment with hesperidin significantly reduced the duration of immobility compared to chronic treatment with vehicle in DM rats (*p <* 0.01 for Hsd-L, *p <* 0.001 for Hsd-H). There were decreases in immobility time of hesperidin (50 mg/kg, 100 mg/kg)-treated groups in age-matched rats, but no statistical significance. Long-term treatment with TBHQ markedly reduced the duration of immobility in diabetic rats (*p <* 0.001; **Figure 1A**). Furthermore, long-term treatment with hesperidin did not change the locomotor counts in the locomotor activity test, which excluded false-positive or false-negative results of hesperidin on depression-like behaviors in the diabetic rats (**Figure 1B**). We also found that long-term hyperglycemia caused a remarkable elevation in corticosterone levels (*p <* 0.001; **Figure 1C**), which was reversed by long-term treatment with hesperidin (*p <* 0.05 for Hsd-L; *p <* 0.001 for Hsd-H; **Figure 1C**).

**Figure 2:**
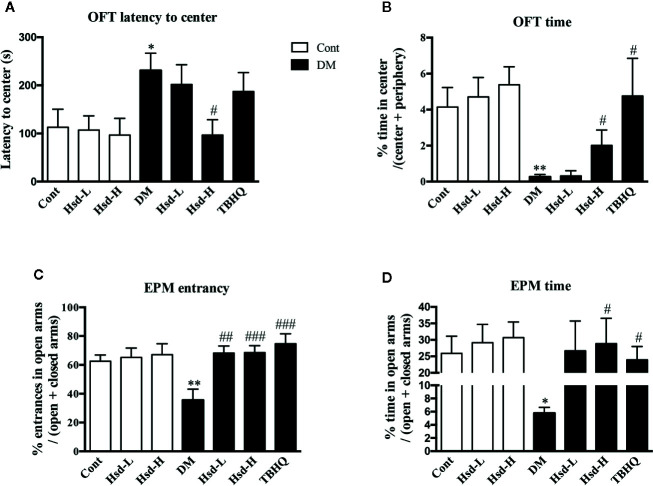
Effects of hesperidin on the anxiolytic-like behaviors of diabetic rats in the open field test (OFT) and elevated plus maze (EPM). **(A)** Latency time to the center area of the OFT. **(B)** Time spent in the center area of the OFT. **(C)** Entrance into the open arms of the EPM. **(D)** Time spent in the open arms of the EPM. Cont, DM, Hsd-L, Hsd-H, and tert-butylhydroquinone (TBHQ) represent the control rats, streptozotocin (STZ)-induced diabetic rats (DM), control or diabetic rats treated with hesperidin at low and high (50, 150 mg/kg) doses, and diabetic rats treated with the Nrf2 activator TBHQ (25 mg/kg), respectively. The results are presented as the mean ± S.E.M., n = 10–11. ^*^p < 0.01, ^**^p < 0.01, *vs*. Cont group; *^#^p* < 0.05, *^##^p* < 0.01, *^###^p* < 0.001, *vs*. DM group.

The data of OFT is presented as **Figures 2A, B**. Two-way ANOVA of the latency to center area showed a major effect on hesperidin treatment factor [F(2, 54) = 3.913, *p <* 0.05] and diabetic factor [F(1, 54) = 9.72, *p <* 0.01]. There was also a significant interaction [F(2,54) = 4.92, *p <* 0.05] between factors. Tukey’s multiple comparisons test showed that the increased latency to the center area of the diabetic rats in the OFT was reversed by chronic treatment with high dose of hesperidin (*p <* 0.05; **Figure 2A**). Moreover, two-way ANOVA revealed significant differences for drug treatment factor [F(2, 54) = 4.40, *p <* 0.05, and diabetic factor (F(1, 54) = 21.59, *p <* 0.001] of time spent in center area, but there was no significance for interactions [F(1, 54) = 1.903, *p >* 0.05]. Hesperidin increased the duration that the diabetic rats stayed in the central area (*p <* 0.05, **Figure 2B**). The Nrf2 inducer TBHQ also increased the time spent in the central area in the OFT (*p <* 0.05, **Figure 2B**). Long-term treatment of hesperidin did not influence the performance of OFT in normal rats.

In the EPM test, as indicated in **Figure 2C**, two-way ANOVA revealed a major effect of hesperidin treatment on the percentage of entries into the open arms [F(2,54) = 5.60; *p <* 0.01], but not on diabetic condition [F(1,54) = 1.30; *p >* 0.05]. In addition, no interaction between factors was detected [F(2,54) = 2.51; *p >* 0.05]. Two-way ANOVA also applied to the data of the time spent in the open arms showed a main effect of hesperidin treatment [F(2,54) = 4.12; *p* < 0.05], but not of diabetic condition [F(1,54) = 2.59; *p >* 0.05]. In addition, no significant interaction between factors was detected [F(2,54) = 2.309; *p >* 0.05]. As shown in **Figures 2C, D**, the diabetic rats spent less time in the open arms, as evidenced by the decreased levels of the percentage of entries into the open arms and the time spent in the open arms (*p <* 0.01 for entries; *p <* 0.05 for time). However, chronic treatment with hesperidin reversed these changes in the EPM test compared to DM group.

### Hesperidin Showed Neuroprotective Effects in the Brains of Diabetic Rats

**Figure 3:**
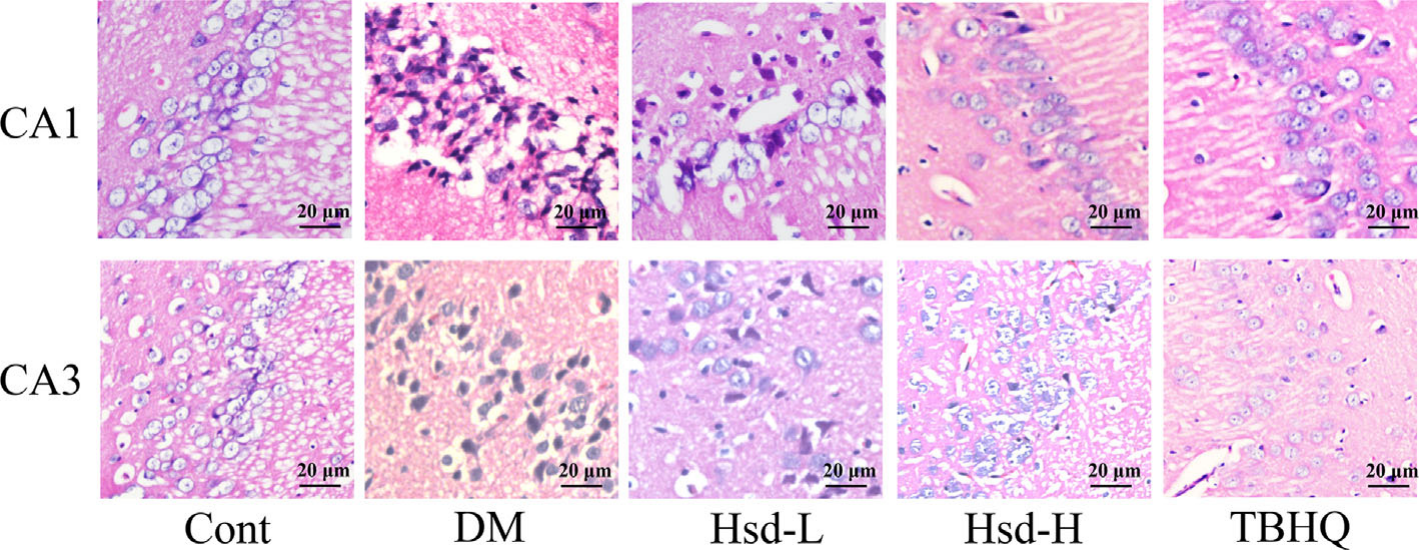
Effects of hesperidin on the changes in neuronal pathomorphology in the hippocampus CA1 and CA3 subfield of diabetic rats (HE ×400, Scale bar = 20 μm). Cont, DM, Hsd-L, Hsd-H, and tert-butylhydroquinone (TBHQ) represent the control rats, streptozotocin (STZ)-induced diabetic rats (DM), diabetic rats treated with hesperidin at low and high (50, 150 mg/kg) doses, and diabetic rats treated with the Nrf2 activator TBHQ (25 mg/kg), respectively.

We used HE staining to observe the pathological changes in the hippocampus of the diabetic rats. The morphological neuronal structure of the CA1 and CA3 subfield of the hippocampus was intact and clear in the vehicle-treated group. In contrast, the neurons in the diabetic group showed prominent karyopycnosis. Chronic treatment with hesperidin and TBHQ ameliorated neuronal karyopycnosis both in the CA1 and CA3 subfield of hippocampus (**Figure 3**).

### Hesperidin Enhanced Glo-1 in the Brains of Diabetic Rats

**Figure 4:**
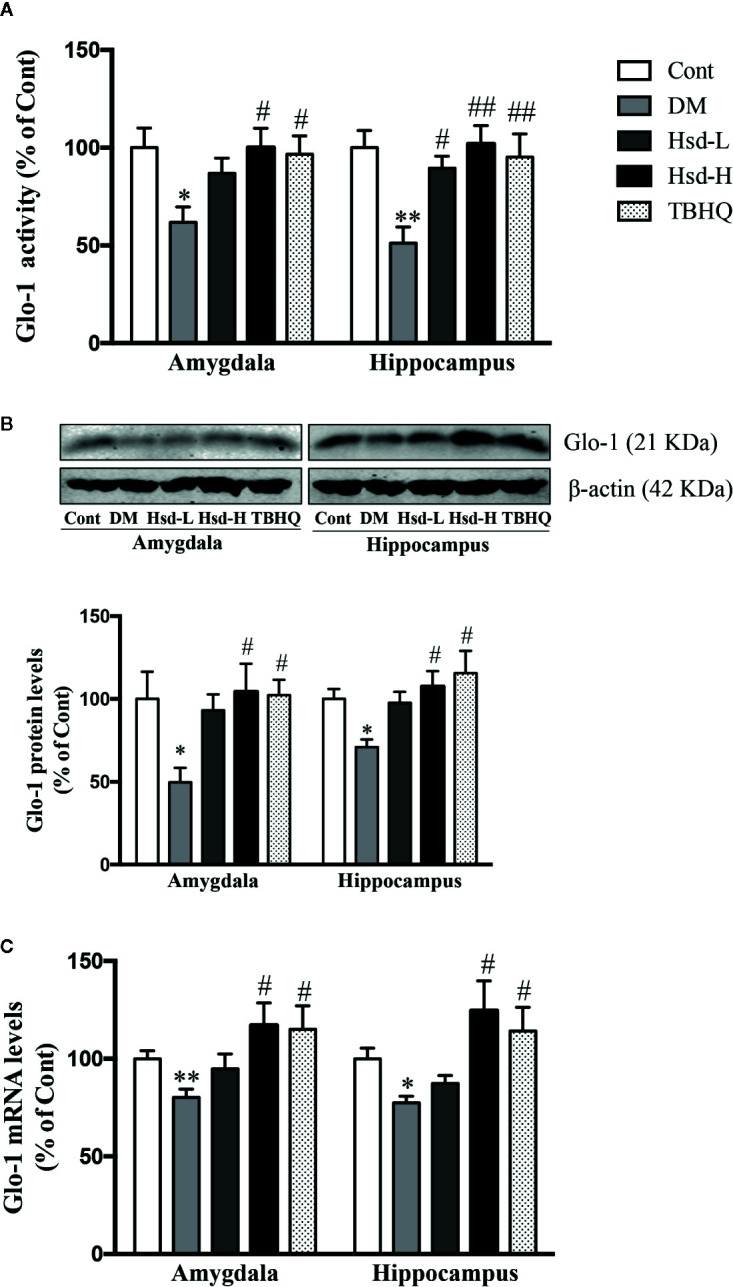
Effects of hesperidin on the Glo-1 activity **(A)**, protein levels **(B)**, and messenger RNA (mRNA) **(C) l**evels in the amygdala and hippocampus of control rats (Cont), streptozotocin (STZ)-induced diabetic rats (DM), diabetic rats treated with hesperidin at low and high (50, 150 mg/kg) doses, and diabetic rats treated with the Nrf2 activator tert-butylhydroquinone (TBHQ) (25 mg/kg). The results are presented as the mean ± S.E.M., n = 7 [activity and messenger RNA (mRNA)], n = 6 (protein). ^*^p < 0.05, ^**^p < 0.01, *vs*. Cont group; *^#^p* < 0.05, *^##^p* < 0.01, *vs*. DM group.

The enzymatic activity, protein levels, and mRNA levels of Glo-1 were examined to investigate its role in the neuroprotective function of hesperidin in type 1 diabetes. We found that the activity, protein levels, and mRNA levels of Glo-1 were decreased in the amygdala and hippocampus of the diabetic rats compared with the normal rats using Dunnett’s multiple comparisons (*p <* 0.05 or *p <* 0.01; **Figures 4A–C**). A high dose of hesperidin clearly increased the Glo-1 activity (*p <* 0.05 for amygdala; *p <* 0.01 for hippocampus; **Figure 4A**), protein levels (both *p <* 0.05; **Figure 4B**), and mRNA levels (both *p <* 0.05; **Figure 4C**) in the diabetic rats, and a low dose of hesperidin only increased the Glo-1 activity in the hippocampus of the diabetic rats (*p <* 0.05; **Figure 4A**). TBHQ also ameliorated all the above indexes (*p <* 0.05 or *p <* 0.01; **Figures 4A–C**).

### Hesperidin Inhibited the AGEs/RAGE Axis in the Brains of Diabetic Rats

**Figure 5:**
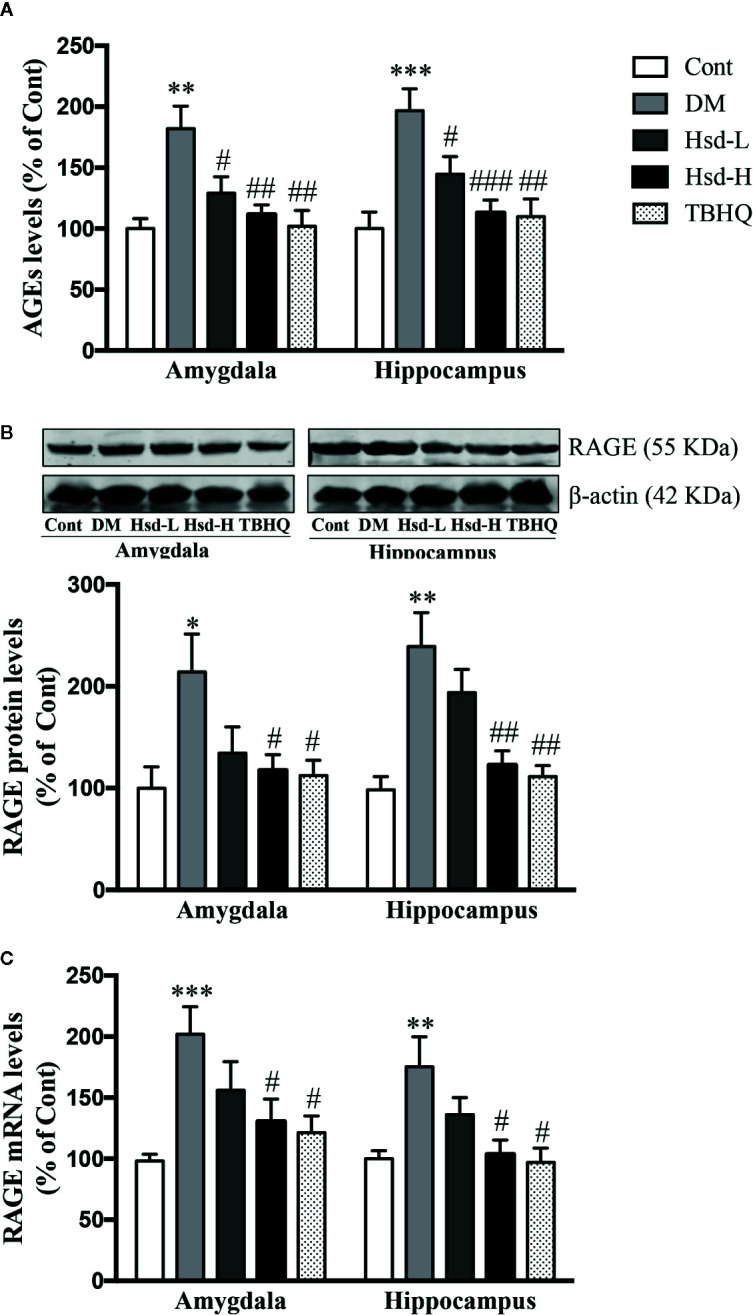
Effects of hesperidin on AGE production **(A)** and protein expression **(B)** and messenger RNA (mRNA) level **(C)** of the receptor for AGEs (RAGE) in the amygdala and hippocampus of control rats (Cont), streptozotocin (STZ)-induced diabetic rats (DM), diabetic rats treated with hesperidin at low and high (50, 150 mg/kg) doses, and diabetic rats treated with the Nrf2 activator tert-butylhydroquinone (TBHQ) (25 mg/kg). The results are presented as the mean ± S.E.M., n = 7 (AGEs, RAGE mRNA level), n = 6 (RAGE protein expression). ^*^p < 0.05, ^**^p < 0.01, ^***^p < 0.001, *vs*. Cont group; *^#^p* < 0.05, *^##^p* < 0.01, *^###^p* < 0.001, *vs*. DM group.

AGEs formation is one of the most important causes of diabetic brain disorders. To study the effect of hesperidin on the AGEs/RAGE axis, we measured the AGEs levels and RAGE protein and mRNA levels in the amygdala and hippocampus of the diabetic rat brains. The AGEs levels and RAGE protein and mRNA levels were dramatically increased in the amygdala and hippocampus of the diabetic rats compared with those of the normal rats (*p <* 0.05, *p <* 0.01, or *p <* 0.001), but a high dose of hesperidin or TBHQ restored these changes in the AGEs and RAGE protein and mRNA levels (**Figure 5**).

### Hesperidin Inhibited Oxidative Stress in the Brains of Diabetic Rats

**Figure 6:**
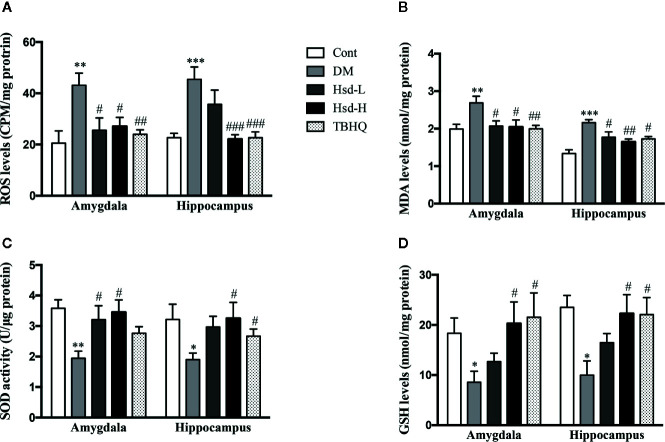
Effects of hesperidin on the reactive oxygen species (ROS) **(A)** and malondialdehyde (MDA) **(B)** levels, superoxide dismutase (SOD) activity, **(C)** and reduced glutathione (GSH) levels **(D)** in the amygdala and hippocampus of control rats (Cont), streptozotocin (STZ)-induced diabetic rats (DM), diabetic rats treated with hesperidin at low and high (50, 150 mg/kg) doses, and diabetic rats treated with the Nrf2 activator tert-butylhydroquinone (TBHQ) (25 mg/kg). The results are presented as the mean ± S.E.M., n = 7. ^*^p < 0.05, ^**^p < 0.01, ^***^p < 0.001, *vs*. Cont group; *^#^p* < 0.05, *^##^p* < 0.01, *^###^p* < 0.001, *vs*. DM group.

To determine whether hesperidin could regulate the levels of oxidative stress in the amygdala and hippocampus of the diabetic rats, the intracellular production of ROS was determined. MDA, one of the well-known end-products of lipid peroxidation, can be used as an indicator of oxidative stress-induced damage. Our results suggested that the ROS and MDA levels were remarkably increased in the amygdala and hippocampus of the diabetic rats (*p <* 0.01 or *p <* 0.001; **Figures 6A, B**), while chronic treatment with hesperidin and TBHQ decreased the ROS and MDA levels (*p <* 0.05, *p <* 0.01, or *p <* 0.001; **Figures 6A, B**). As shown in **Figures 6C, D**, we also measured the activity of SOD and the levels of GSH, two common endogenous antioxidants, in hippocampal and amygdala neurons. In addition, GSH is also a cofactor for Glo-1 activity. The changes in these indexes reflect the strength of the cellular antioxidant capacity and the degree of oxidative injury. Our data showed that the SOD activity and GSH levels were both decreased in the amygdala and hippocampus of the diabetic rats (*p <* 0.05 or *p <* 0.01). The SOD activity and GSH levels in the diabetic group were restored to nearly normal levels after chronic treatment with hesperidin. The Nrf2 activator TBHQ reversed the decreased SOD activity and GSH levels in the hippocampus and the decline GSH level in the amygdala of the diabetic rats (all *p <* 0.05; **Figures 6C, D**).

### Hesperidin Activated Nrf2/ARE Signaling in the Brains of Diabetic Rats

**Figure 7:**
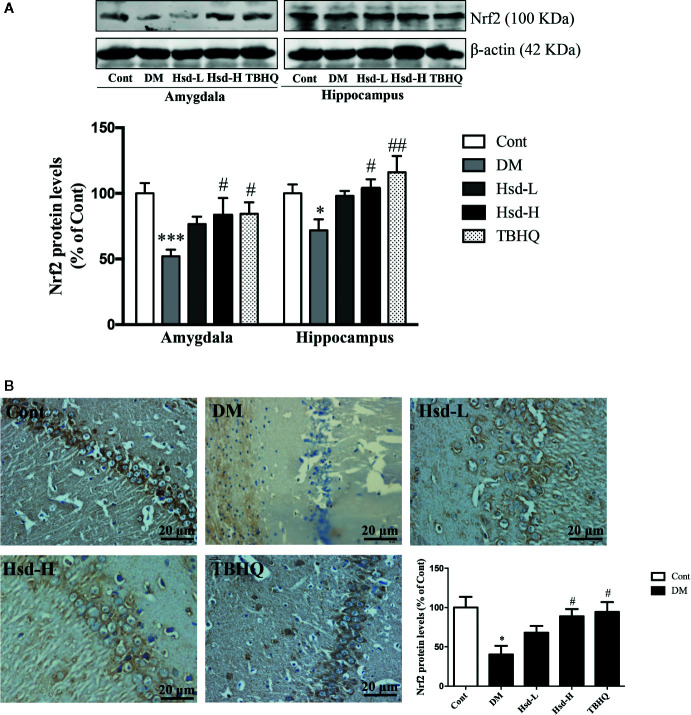
Effects of hesperidin on the protein levels of Nrf2 assessed by Western blotting **(A)** in the amygdala and hippocampus and by immunohistochemistry **(B)** in the hippocampus of control rats (Cont), streptozotocin (STZ)-induced diabetic rats (DM), diabetic rats treated with hesperidin at low and high (50, 150 mg/kg) doses, and diabetic rats treated with the Nrf2 activator tert-butylhydroquinone (TBHQ) (25 mg/kg). The results are presented as the mean ± S.E.M., n = 6 (protein), n = 4 (IHC). ^*^p < 0.05, ^***^p < 0.001, *vs*. Cont group; *^#^p* < 0.05, *^##^p* < 0.01, *vs*. DM group.

**Figure 8:**
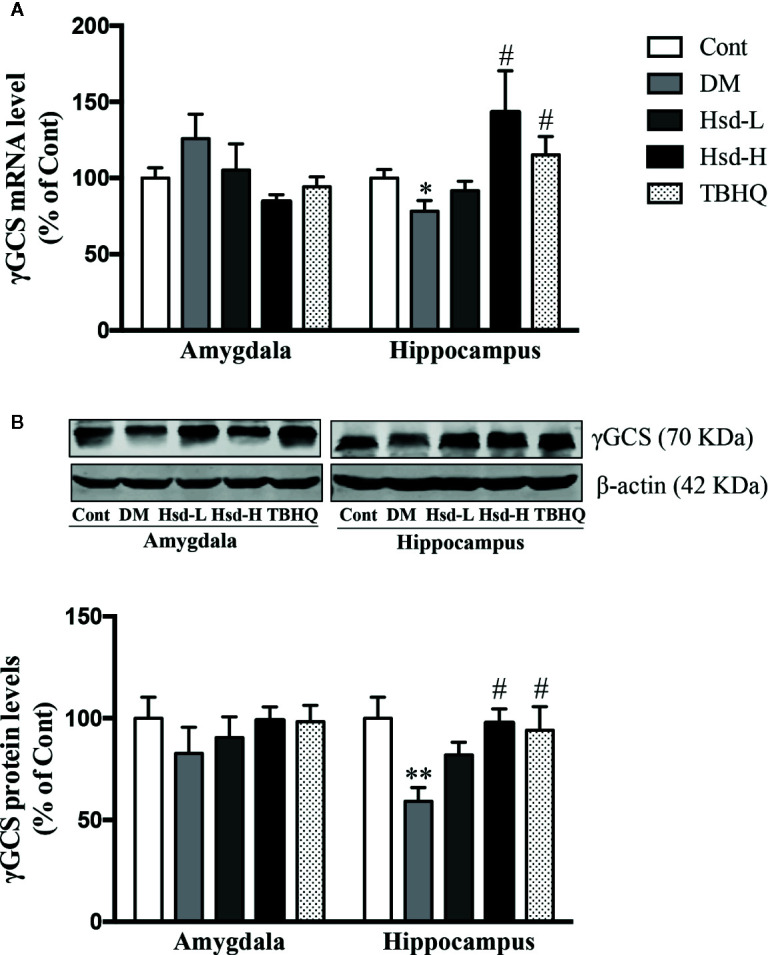
Effects of hesperidin on the γ-glutamylcysteine synthetase (γ-GCS) messenger RNA (mRNA) **(A)** and protein **(B)** levels in the amygdala and hippocampus of control rats (Cont), streptozotocin (STZ)-induced diabetic rats (DM), diabetic rats treated with hesperidin at low and high (50, 150 mg/kg) doses, and diabetic rats treated with the Nrf2 activator TBHQ (25 mg/kg). The results are presented as the mean ± S.E.M., n = 7 (mRNA), n = 6 (protein). ^*^p < 0.05, ^**^p < 0.01, *vs*. Cont group; *^#^p* < 0.05, *vs*. DM group.

To study whether the hesperidin-induced Glo-1 enhancement was caused by the activation of the Nrf2/ARE signaling pathway, the subcellular localization of Nrf2 and the protein and mRNA levels of γ-GCS were detected in the amygdala and hippocampus of the diabetic rats. In addition, the level of GSH was also determined to assess the γ-GCS activity. The Nrf2 levels were significantly decreased in the brains of the diabetic rats (*p <* 0.001 for amygdala; *p <* 0.05 for hippocampus; **Figure 7A**), but high dose of hesperidin and TBHQ reversed this decrease in the Nrf2 levels (*p <* 0.05 or *p <* 0.01; **Figure 7A**). In addition, compared with diabetic rats, hesperidin at a high dose and TBHQ increased Nrf2 levels in the nucleus (*p <* 0.05; **Figure 7B**). The activation of the Nrf2/ARE signaling pathway by hesperidin was further assessed by evaluating changes in its downstream molecules. The γ-GCS, a downstream target regulated by Nrf2/ARE signaling was elevated, in terms of its protein expression and mRNA levels in the hippocampus of the diabetic rats (*p <* 0.05, *p <* 0.01, respectively; **Figures 8A, B**). However, the protein and mRNA levels of γ-GCS were not significantly changed in the amygdala of the diabetic rats.

### Neuroprotective Effects of Hesperidin in N2a Cells

**Figure 9:**
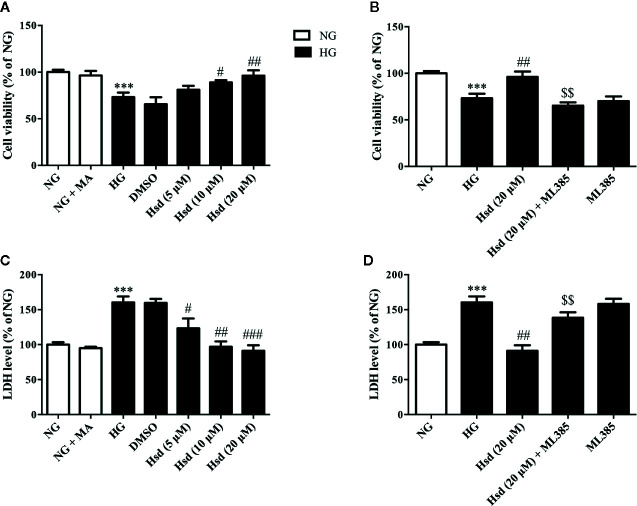
The protective effects of hesperidin against high glucose (HG)-induced neurotoxicity in N2a cells. **(A)** Effects of hesperidin on the viability of N2a cells. **(B)** Treatment with ML385, an Nrf2 inhibitor, blocked the neuroprotective effects of hesperidin on the viability of HG-treated cells. **(C)** Effects of hesperidin on the LDH release from N2a cells. **(D)** Treatment with ML385, an Nrf2 inhibitor, blocked the neuroprotective effects of hesperidin on the LDH release of HG-treated cells. The results are presented as the mean ± S.E.M., n = 6. ^***^ *p* < 0.001, *vs*. NG group. *^#^p* < 0.05, *^##^p* < 0.01 and ^###^ *p* < 0.001 *vs*. the DM group. *^$$^*p < 0.01 *vs*. Hsd (20 μM) group.

*In vitro*, mannitol was used as an osmotic control to eliminate the effect of an osmotic change induced by HG, and DMSO served as the solvent control for hesperidin or ML385. We examined the effects of mannitol and DMSO on the viability and LDH release of N2a cells. We found that mannitol had no effect on the N2a cell viability and the LDH levels released compared to normal glucose (**Figures 9A, C**), indicating that the osmotic pressure induced by high glucose did not affect the experimental results. The results in the HG + DMSO group were almost identical to those in the HG group, demonstrating that the presence of DMSO did not affect the experimental data.

The MTT assay results revealed that high glucose obviously reduced the viability of N2a cells compared with normal glucose (*p <* 0.001; **Figure 9A**). However, this change in viability was restored by hesperidin at doses of 10 and 20 μM for 48 h (*p <* 0.05 and *p <* 0.01). Neuronal damage was also quantitatively assessed by measuring the activity of the LDH released from the N2a cells. The results revealed significantly increased LDH release under high-glucose conditions (*p <* 0.001; **Figure 9C**). However, this increase was reversed by treatment with hesperidin to different degrees. The Nrf2 inhibitor ML385 at 10 μM prevented hesperidin at 20 μM from inducing an increase in cell viability and a decrease in LDH release in high glucose-treated cells (both *p <* 0.01; **Figures 9B, D**). Notably, ML385 decreased the cell viability and increased the LDH levels, and the addition of high levels of glucose did not cause additional effects, indicating that the inhibition of Nrf2 specifically plays a role in the neuroprotective effects of hesperidin.

### Hesperidin Activated Nrf2/ARE Signaling in N2a Cells Cultured With High Glucose

**Figure 10:**
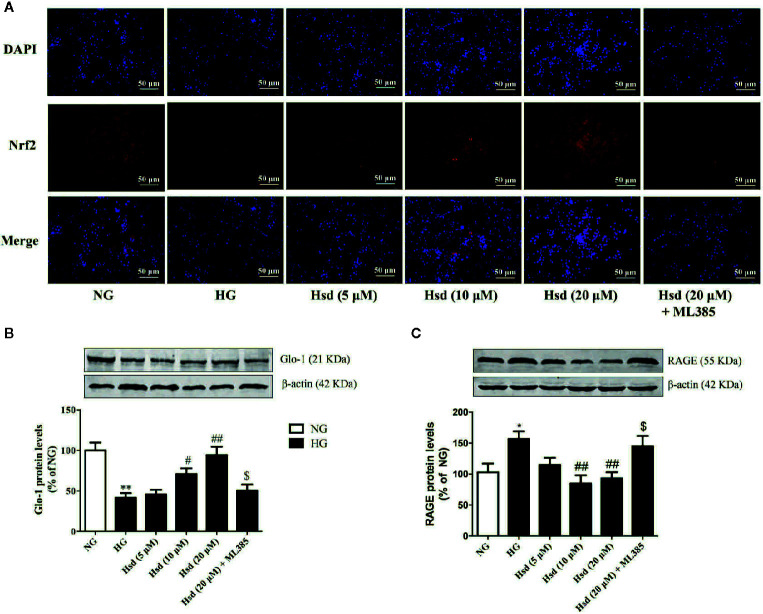
**(A)** Effects of hesperidin on Nrf2 expression detected by immunofluorescence in N2a cells cultured with high glucose. **(B)** Effects of hesperidin on Glo-1 levels in N2a cells cultured with high glucose. **(C)** Effects of hesperidin on RAGE protein expression in N2a cells cultured with high glucose. The results represent the mean ± S.E.M., n = 3. ^*^ *p* < 0.05 and ^**^ *p* < 0.01, *vs*. NG group. ^#^ *p* < 0.05 and ^##^ *p* < 0.01, *vs*. DM group. ^$^ *p* < 0.05 *vs*. Hsd (20 μM) group.

To characterize the Nrf2/ARE signaling pathway responsible for the protective effects of hesperidin on N2a cells chronically cultured with high glucose, the Nrf2 inhibitor ML385 was used to observe the changes in Nrf2, Glo-1, and RAGE protein expression. The results showed that Nrf2 expression was markedly decreased in the N2a cells cultured with high glucose, according to the immunofluorescence method (**Figure 10A**). However, this decrease was reversed by treatment with hesperidin for 48 h. When the cells were treated with the Nrf2 inhibitor ML385, the effects of hesperidin on Nrf2 were prevented.

We also investigated Glo-1 and RAGE protein expression in the presence of hesperidin in N2a cells treated with high glucose. As shown in **Figures 10B, C**, the high glucose-induced decrease in Glo-1 expression and increase in RAGE levels were reversed by hesperidin at middle and high doses (*p <* 0.05 or *p <* 0.01). However, these effects were reversed by treatment with ML385 (both *p <* 0.05), suggesting that Nrf2 participates in the neuroprotective effects of hesperidin against high glucose-induced damage.

## Discussion

The prevalence rates of depression could be up to three-times higher in type 1 diabetic patients and twice as high in type 2 diabetic patients compared with general population worldwide (Roy and Lloyd, 2012). On the other hand, depression may increase the risk of developing type 2 diabetes with 60% (Mezuk et al., 2008). So development of novel antidepressants with anti-diabetic mechanisms of action would provide a novel insight into drug discovery. Evidence verifies that the induction of Glo-1 leads to clear prophylactic and therapeutic effects on diabetic complications (Maher et al., 2011; Jack et al., 2012; Skapare et al., 2012; Liu et al., 2013b; Rabbani and Thornalley, 2014), including diabetic encephalopathy (Liu et al., 2013a; Liu et al., 2017b; Zhu et al., 2018). In this study, we found that hesperidin exhibited antidepressant effects *via* the enhancement of Glo-1 function in the amygdala and hippocampus of diabetic rats, which led to a decrease in the formation of AGEs and damage due to oxidative stress. Moreover, the upregulation of Glo-1 by hesperidin was related to the activation of the Nrf2/ARE signaling pathway, which further elucidated the molecular mechanism by which hesperidin inhibited AGE formation and oxidative stress in the brains of diabetic rats. Hesperidin also showed protective effects on the cell lesions caused by high glucose, as evidenced by increased cell viability in the MTT assay and decreased LDH release levels. Further *in vitro* studies suggested that treatment with hesperidin reversed the HG-induced decrease in Nrf2 expression. This effect is supported by a pharmacological interaction study that suggested that the Nrf2 inhibitor ML385 reversed the neuroprotective effects of hesperidin against cell lesions. These findings provide evidence that the antidepressant-like effects of hesperidin are predominantly mediated by activating the Nrf2/ARE/Glo-1 pathway.

The antidepressant-like effects of hesperidin have been reported in both forced swimming and tail suspension tests, which are used to evaluate depression (Souza et al., 2013). Furthermore, hesperidin reduced the depression-like behaviors in the tail suspension test induced by intrastriatal injection of 6-hydroxydopamine in an animal model of Parkinson’s disease (Antunes et al., 2014). The anxiolytic effect of hesperidin following oral administration has also been reported in the elevated plus-maze and mirror chamber tests of anxiety (Viswanatha et al., 2012). This study showed that in diabetic rats, hesperidin exerted antidepressant effects as evidenced by decreased immobility time in the FST, anxiolytic effects as evidenced by increased time spent in the center area of the OFT, and increased percentage of entries into the open arms and time spent in the open arms in the EPM. Previous investigations revealed that hesperidin exerted neuroprotective effects both *in vitro* and *in vivo*. For example, hesperidin had neuroprotective effects on 3-nitropropionic acid-induced (Menze et al., 2012), amyloid β-induced (Huang et al., 2012), and H_2_O_2_-induced (Choi and Ahn, 2008) neurotoxicity. In our study, hesperidin showed neuroprotective effects against hyperglycemia-induced neuronal damage in the hippocampus, as evidenced by an increased number of healthy cells in the CA1 and CA3 areas of the hippocampus. Moreover, hesperidin exerted cytoprotective effects against amyloid β-induced damage to glucose transport in Neuro-2A cells by downregulating neuronal autophagy (Huang et al., 2012). These results suggested that hesperidin exerted clear antidepressant and anxiolytic effects in diabetic rats.

The antidepressant and anxiolytic effects of hesperidin are partly mediated by its enhancement of Glo-1 function in the amygdala and hippocampus of diabetic rats. The glyoxalase system plays an important role in astrocyte-mediated neuroprotection (Belanger et al., 2011). AGEs, mitochondrial dysfunction, and the glyoxalase system, specifically Glo-1, play a role in the progression and modulation of diabetic peripheral neuropathy (Jack et al., 2012). Many natural products, such as hesperetin, fisetin, mangiferin, and resveratrol, are reported to increase the expression and activity of Glo-1 (Maher et al., 2011; Liu et al., 2013a; Liu et al., 2013b; Xue et al., 2016; Liu et al., 2017b). This study revealed that hesperidin significantly increased the enzymatic activity, protein levels, and mRNA levels of Glo-1 in the amygdala and hippocampus of diabetic rats, and these increases were accompanied by elevated levels of GSH, an integrant cofactor of Glo-1 activity, indicating that the induction of Glo-1 may contribute to the neuroprotective effects of hesperidin. Glo-1 is known to rapidly remove MG and decrease the formation and accumulation of AGEs, since MG is a main precursor of AGEs formation (Shinohara et al., 1998). Furthermore, Glo-1 can also directly inhibit AGEs formation in endothelial cells (Thornalley, 2003). In fact, Glo-1 overexpression could restore the increases in the levels of AGEs and RAGE caused by hyperglycemia in diabetic rats (Brouwers et al., 2011). In this study, we found that hesperidin reversed the elevated levels of AGEs and RAGE mRNA and protein expression in the brains of diabetic rats, which offered further proof of the hesperidin-induced enhancement of Glo-1. Interestingly, hesperidin was found to possess relatively strong inhibitory activity against AGEs formation (Li et al., 2012), revealing a potential direct inhibitory effect of hesperidin on the formation of AGEs in diabetes.

There is growing evidence that suggests that oxidative stress is a central mechanism for the pathogenesis of diabetic complications (Reagan et al., 2000). We and others have demonstrated that the oxidative stress caused by hyperglycemia plays a vital role in the development of diabetic complications, including diabetic encephalopathy (Kuhad and Chopra, 2007; Liu et al., 2012; Liu et al., 2013a; Zhu et al., 2018). AGEs elevate ROS production and injure antioxidant systems, and some AGEs formation is induced under oxidative conditions in diabetes, producing a vicious cycle (Nowotny et al., 2015). Moreover, Glo-1 overexpression decreased the hyperglycemia-induced elevation of oxidative stress and AGEs levels in diabetic rats (Brouwers et al., 2011). Elevated Glo-1 levels could reduce oxidative stress and AGEs accumulation in the brains of diabetic rats (Liu et al., 2013a; Zhu et al., 2018). These studies suggested that oxidative stress and protein glycation could play vital roles in the pathogenesis of emotional disorder in diabetic rats, and Glo-1 may be a potential target for the prevention and treatment of diabetic complications. We found a remarkable elevation in oxidative stress in the amygdala and hippocampus of diabetic rats, which was reflected by elevated levels of ROS and MDA (an important biomarker of lipid peroxidation), reduced levels of GSH (a powerful endogenous antioxidant that is the first line of defense against free radicals), and decreased activity of SOD (a potent endogenous radical scavenger), indicating that Glo-1 downregulation may lead to increased oxidative stress. As expected, chronic treatment with hesperidin significantly improved the aforementioned alterations caused by chronic hyperglycemia, as it restored the ROS, MDA, SOD, and GSH levels in the brain to levels similar to those observed in TBHQ-treated rats. This finding is consistent with prior studies by Liu et al. (2017a) and Mahmoud et al. (2012), who confirmed the antioxidant activity of hesperidin in diabetic rats. Thus, the inhibitory effect of hesperidin on oxidative stress could be one of the mechanisms responsible for the effect of hesperidin in diabetic rats.

The upregulation of Glo-1 by hesperidin during high glucose-induced neuronal damage is caused by activating the Nrf2/ARE signaling pathway. Nrf2 and Nrf2-mediated phase II antioxidant enzymes are increasingly understood to play important roles in neuroprotection and treatment of neurological diseases (Zhang et al., 2013; Yao H. et al., 2018; Yao H. K. et al., 2019). Glo-1 is a phase II detoxifying enzyme that is regulated and controlled by the Nrf2/ARE signaling pathway (Xue et al., 2012); thus, Glo-1 is similar to γ-GCS, a phase II antioxidant enzyme that is regulated by the Nrf2/ARE pathway. Moreover, our group further confirmed that the inhibition of the Nrf2/ARE signaling pathway could reduce the function of Glo-1 in the primary hippocampal and cerebral cortical neurons of rats chronically exposed to high glucose (Liu et al., 2017b). According to our previous report, Glo-1 was significantly downregulated in the brains of diabetic rats, and the natural products mangiferin and quercetin ameliorated brain damage in diabetic rats through the enhancement of Glo-1 functions (Liu et al., 2013a; Zhu et al., 2018). This study revealed that hesperidin significantly improved the brain damage in diabetic rats, which was accompanied by the activation of Nrf2/ARE signaling, as evidenced by the elevated levels of Nrf2 and the increased mRNA level, protein level, and enzymatic activity (reflected by GSH levels) of γ-GCS. In addition, hesperidin was reported to inhibit H_2_O_2_-induced oxidative stress in hepatocytes through the induction of heme oxygenase 1 (HO-1), which was mediated by the activation of MAPK/Nrf2 signaling (Chen et al., 2010), and to improve inflammatory responses in lipopolysaccharide-stimulated RAW 264.7 cells through the inhibition of NF-κB and the activation of Nrf2/HO-1 signaling (Ren et al., 2016). Taken together, these results illustrate that hesperidin activates the Nrf2/ARE pathway in diabetic rats with depression and anxiety-like behaviors, subsequently upregulating Glo-1.

Subsequently, the *in vitro* study provided promising evidence that the neuroprotective effects of hesperidin occur by activating Nrf2 signaling. Hesperidin restored the HG-induced decreases in cell viability and increases in the levels of released LDH. However, these effects were blocked by the Nrf2 inhibitor ML385. Subsequent biochemical studies suggested that hesperidin prevented the HG-induced decreases in Nrf2 and Glo-1 expression and increases in RAGE expression *in vitro*. However, these effects were prevented by treating N2a cells with Ml385, supporting the idea that Nrf2 is important for the regulation of Glo-1 and RAGE.

In summary, this study demonstrated that hesperidin could alleviate the depression- and anxiety-like behaviors of diabetic rats. The neuroprotective effects of hesperidin, which are mediated through the enhancement of Glo-1 and/or the inhibition of the AGE/RAGE interaction, are due to the activation of the Nrf2/ARE pathway. This study presents a potential clinical application of flavonoid compounds from citrus species in the prevention and treatment of diabetic central neuropathy.

## Data Availability Statement

All datasets generated for this study are included in the article/supplementary material.

## Ethics Statement

The animal study was reviewed and approved by Xuzhou Medical University Committee on Animals Care and Use.

## Author Contributions

XY and YWL supervised the study and analyses. XZ, HL, and YL wrote the manuscript. XZ, YL, and HL performed the behavioral assays and statistical analysis of the data. XZ and YL performed the bioinformatics analyses. YC raised the animals.

## Funding 

This work was supported by the Natural Science Fund for Colleges and Universities in Jiangsu Province (18KJB31008), the Science and Technology Projects of Xuzhou City (KC18203), and the Research Fund of Xuzhou Medical University (2018KJ03).

## Conflict of Interest

The authors declare that the research was conducted in the absence of any commercial or financial relationships that could be construed as a potential conflict of interest.
